# Supplementary material for: BRAFV600E Induction in Thyrocytes Triggers Important Changes in the miRNAs Content and the Populations of Extracellular Vesicles Released in Thyroid Tumor Microenvironment
Source: Biomedicines. 2022 Mar 23;10(4):755. doi: 10.3390/biomedicines10040755 (PMC9029139; doi:10.3390/biomedicines10040755)
Supplement: Supplementary file 1 [file biomedicines-10-00755-s001.zip › biomedicines-1634772-supplementary.pdf]

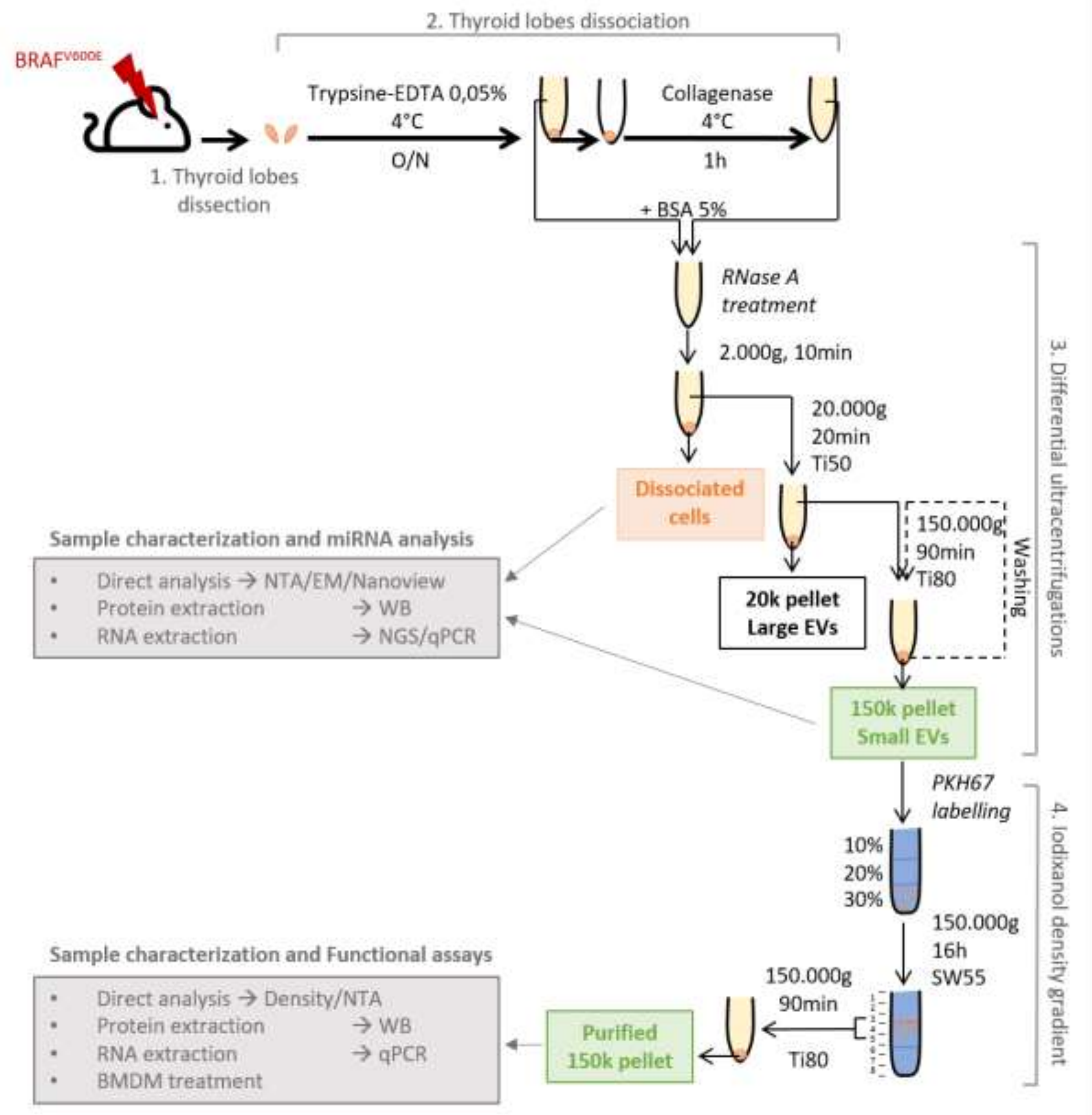

Supplementary Figure S1: In vivo produced EVs are isolated from thyroid lobes by tissue dissociation followed by differential ultracentrifugation and optional iodixanol density gradient. Schematic representation of the procedure for EV isolation, characterization, and downstream analyses.

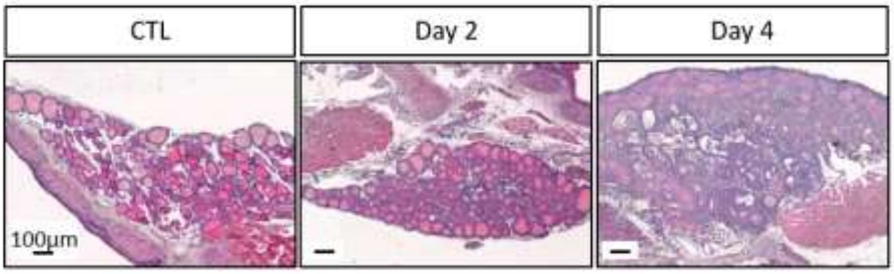

Supplementary Figure S2: BRAF^{V600E} expression in thyrocytes triggers tissue growth and remodelling Low-magnification view of the haematoxylin and eosin staining of thyroid tissues from control and doxycycline-treated mice, after 2 and 4 days of intraperitoneal injections, shown in Fig.1A.

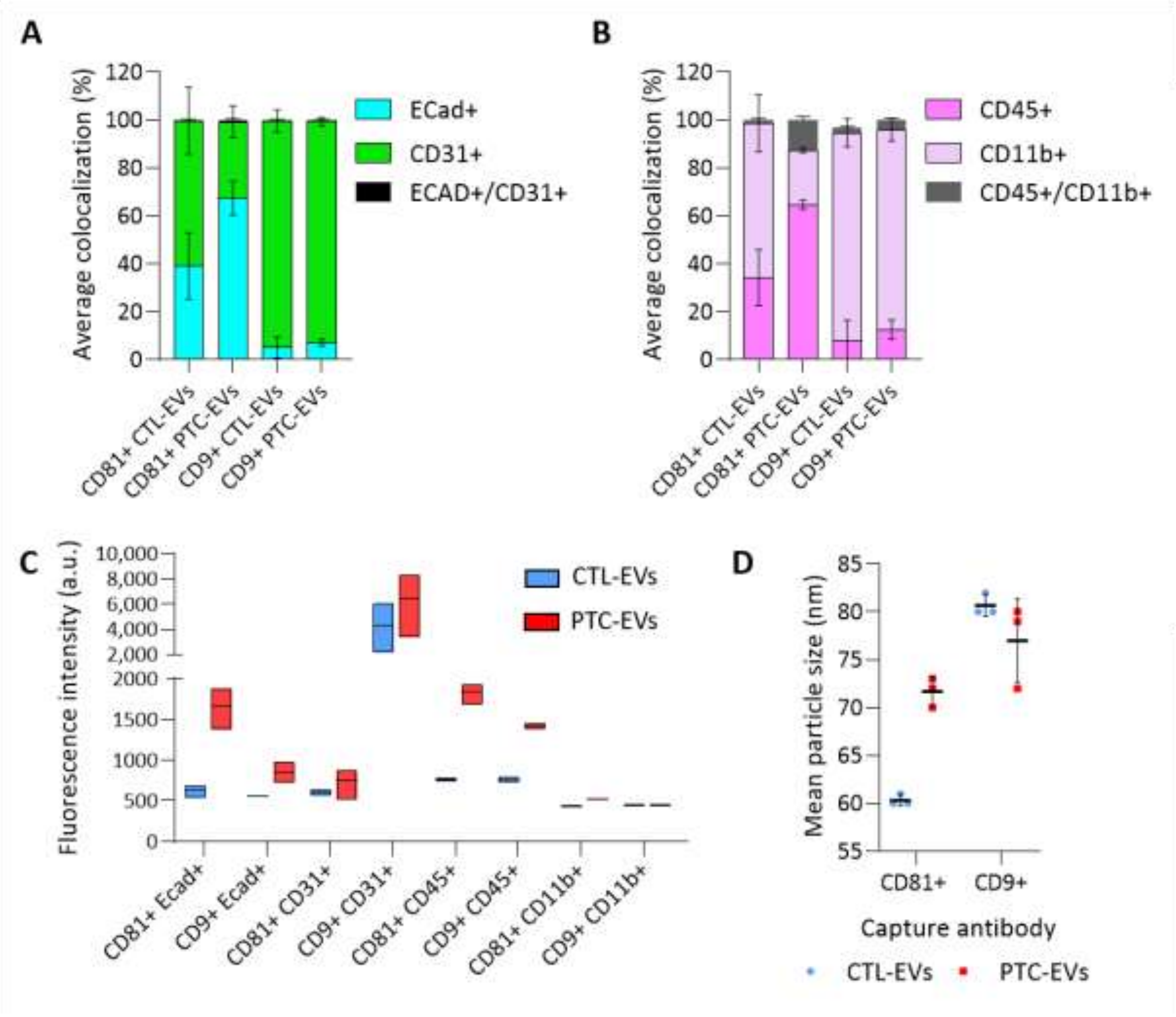

Supplementary Figure S3: The ExoView platform allows EV population analyses. (**A-B**) Average percentage of colocalization between Ecad and CD31 detection antibodies (**A**), and between CD45 and CD11b detection antibodies (**B**). (**C**) Fluorescence intensity of detection antibodies measured in CD9+ and CD81+ EV populations in control and PTC- EV samples, reflecting the number of detected epitopes. (**D**) Mean particles size of CD9+ and CD81+ EVs measured in CTL and PTC-EV samples.

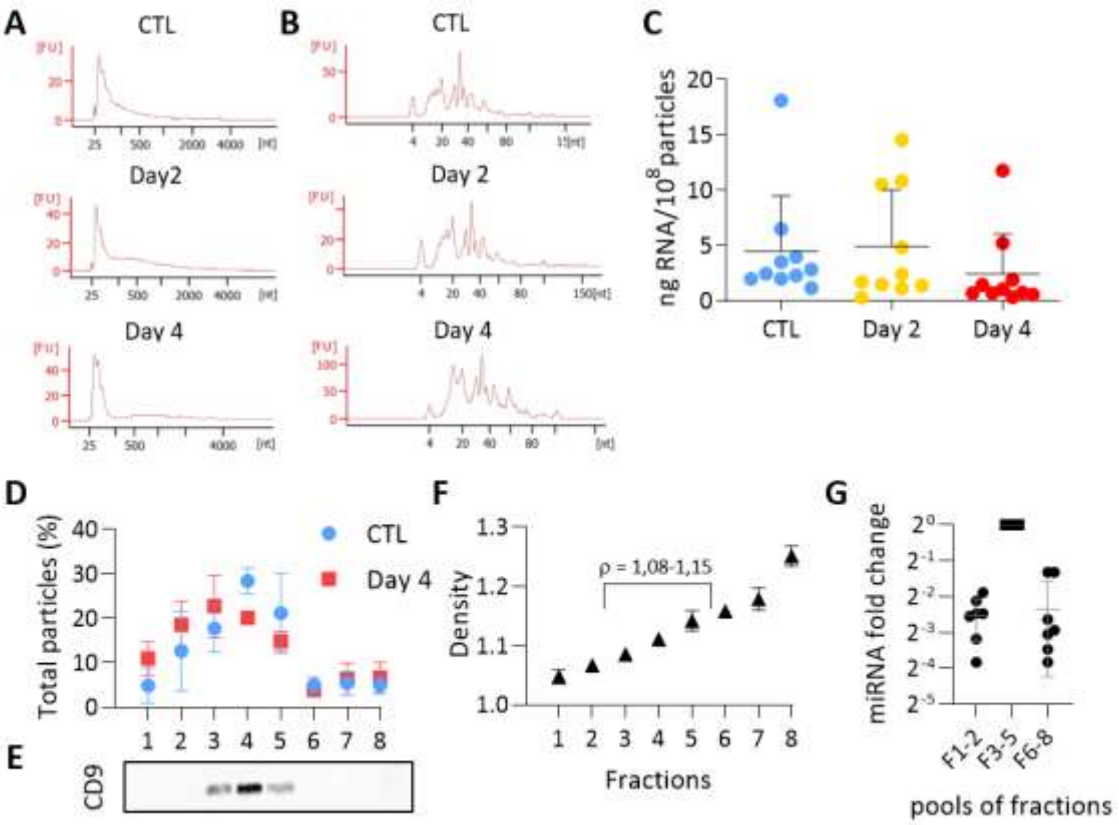

Supplementary Figure S4: miRNAs detected in the high-speed pellets are associated with EV fractions after density-based fractionation. Electrophoretic profiles of (**A**) total RNA, and (**B**) small RNA extracted from the 150k pellet isolated from control and DOX-treated thyroids, after 2 and 4 days of injections. (**C**) Total RNA quantity per 10^8 particles (measured by NTA). (**D-G**) Characterization of 8 fractions obtained after bottom-up iodixanol density gradient. (**D**) Distribution of particles measured by NTA. (**E**) Western blot detection of CD9 marker after purification of the 150k pellet isolated from day 4 DOX-treated thyroids. (**F**) Measure of the density of each fraction. (**G**) RT-qPCR analysis of 8 miRNAs in concentrated pools of fractions. Data are expressed as miRNA fold changes in pools of fractions 1-2 and 6-8 compared to their abundance in pool of fractions 3 to 5, considered as EV fractions.

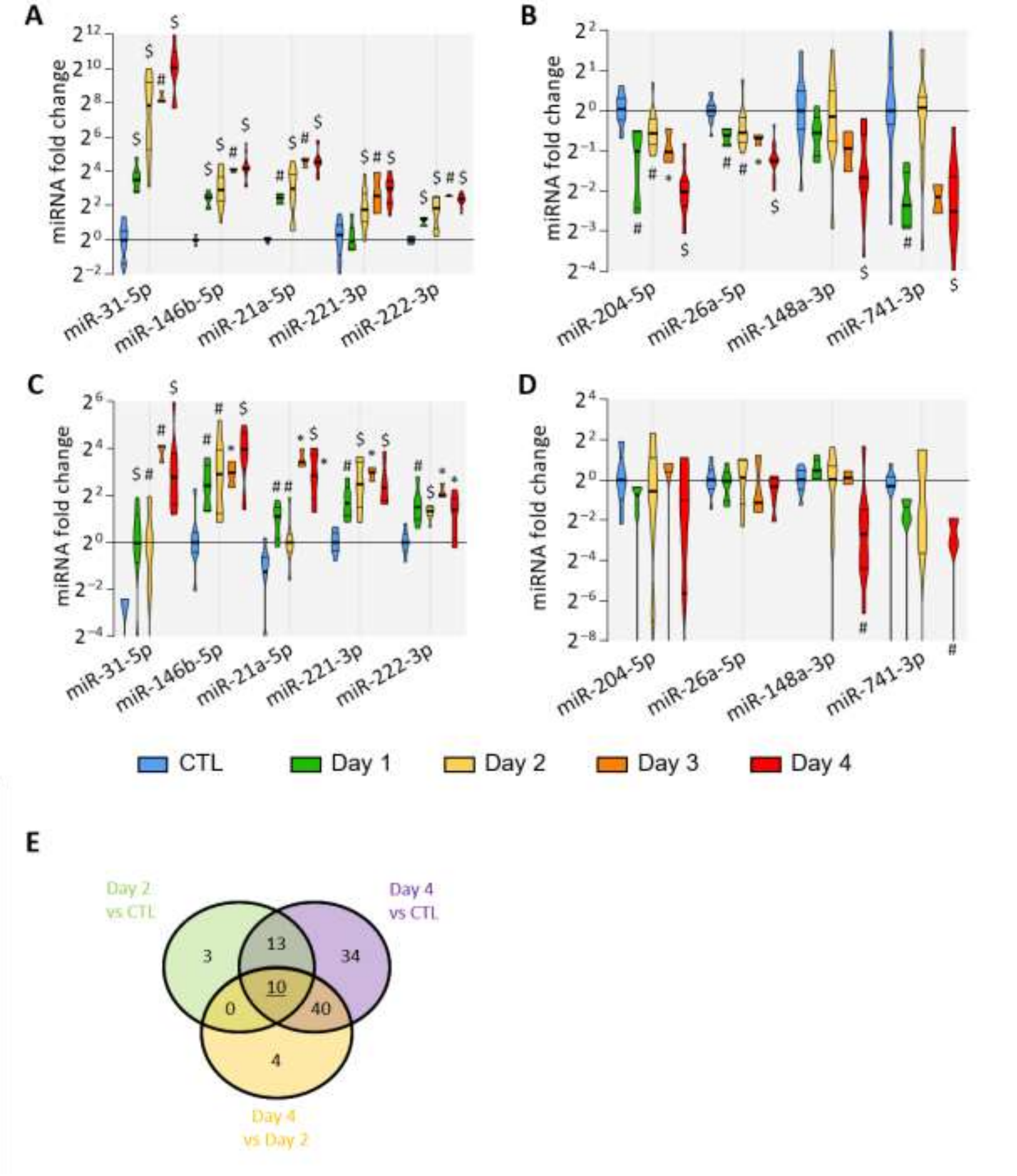

Supplementary Figure S5: miRNAs display rapid and important changes in their abundance in BRAF^{V600E} tissues and EVs. RT-qPCR analyses of upregulated (**A, B**) and downregulated (**C, D**) miRNA in dissociated cells (**A, C**) and EVs (**B, D**) from control and doxycycline-treated thyroids after 1, 2, 3 and 4 days of injections (n≥4). MiRNA fold changes are normalized on let-7c-5p expression for cellular samples, and on the geometric mean of miR-126a-3p and let-7b-5p expression for EV samples. Data are expressed as mean ± SD (* $p<0.05$; # $p<0.01$; $ $p<0.001$). (**E**) Venn diagram illustrating the number of EV-miRNAs with differential abundance in the three groups. Abundance of 10 miRNAs was statistically different between the 3 groups.

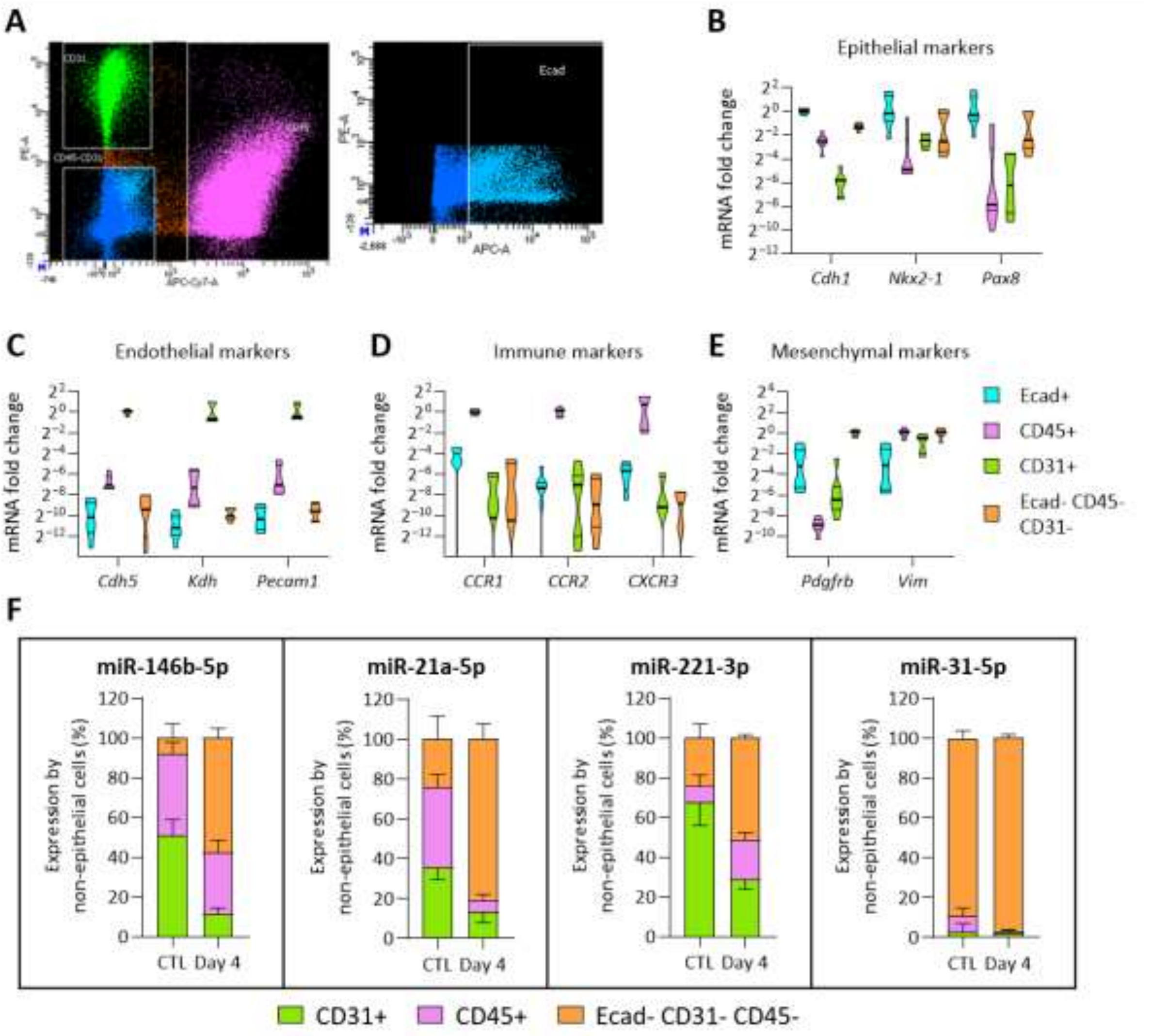

Supplementary Figure S6: Purification of epithelial, endothelial and immune populations by FACS-sorting of thyroid and tumour dissociated cells using Ecad, CD31 and CD45 markers. (**A**) Coloured dot plot showing an example of DOX day 4 dissociated cells sorting according to the intensity of fluorescence with CD31, CD45 or Ecad antibodies. (B-E) RT-qPCR analyses of the expression of epithelial (**B**), endothelial (**C**), immune (**D**) and mesenchymal (**E**) markers in sorted populations. mRNA fold changes are normalized on the geometric mean of *Rpl27* and *Gapdh* expression and compared to the expression in their respective population. (**F**) RT-qPCR analyses of miRNA expression in FACS-sorted non-epithelial cell populations. Stacked bars show the relative expression of the miRNAs in non-epithelial cell populations normalized by the quantity of RNA extracted from each population. Data are expressed as mean ± SD.

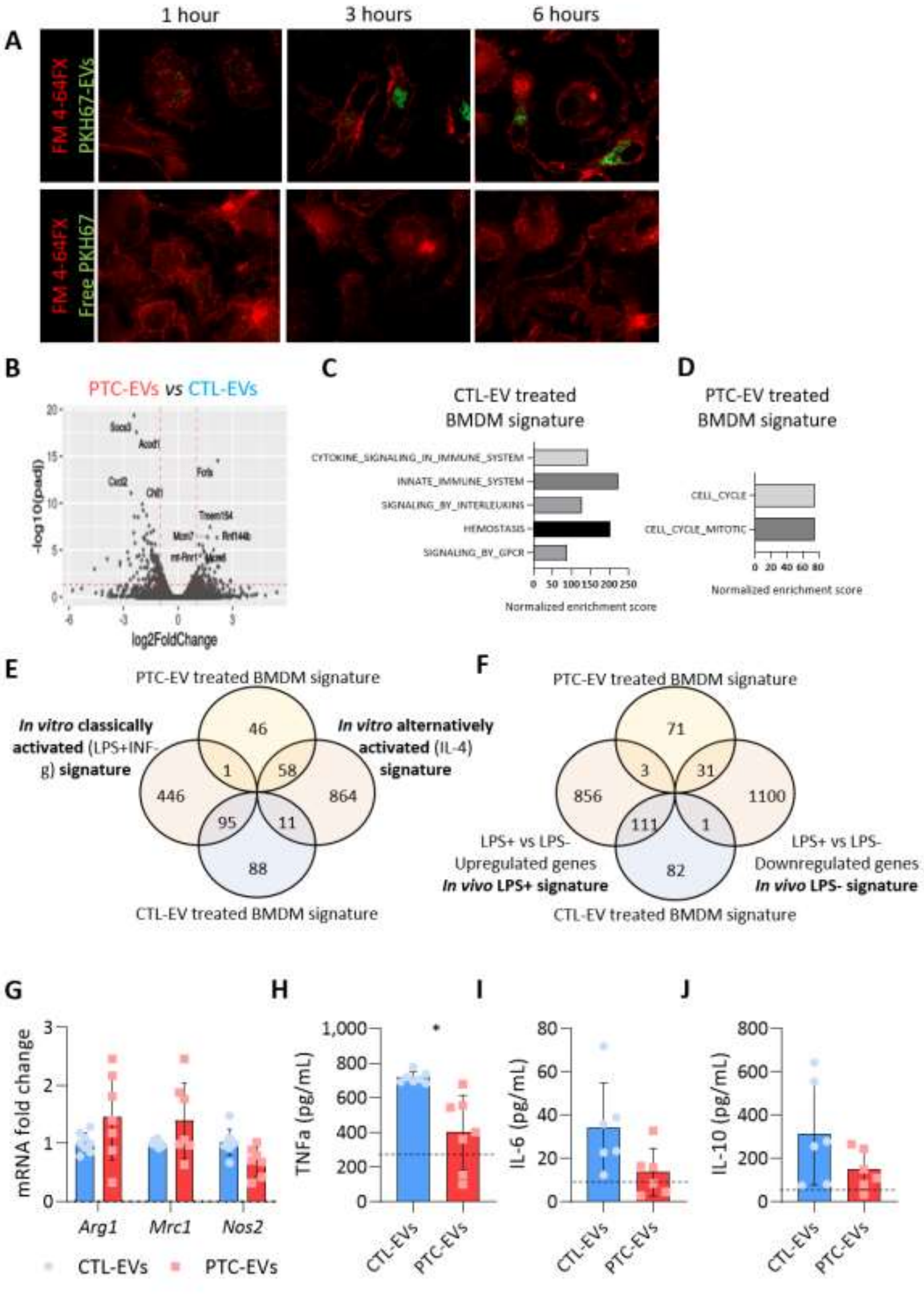

Supplementary Figure S7: Treatment of BMDM with CTL-EVs or PTC-EVs pools demonstrate internalization but no major effects. (**A**) Live imaging of BMDM fluorescently labelled by FM 4-64FX, a red membrane dye, and treated with PKH67-EVs or free PKH67 after 1, 3 or 6 hours of incubation. (**B**) Volcano plot showing p-adjusted value and fold change relation in differential analysis of mRNA

expression by CTL-EVs or PTC-EVs treated BMDM. KEGG pathway analysis of gene upregulated in CTL-EVs treated BMDM compared to PTC-EVs treated BMDM (**C**) and of gene upregulated in PTC-EVs treated BMDM compared to CTL-EVs treated BMDM (**D**). (**E-F**) Comparison of the mRNA signature of EV-treated BMDM with publicly available mRNA signature of (**E**) *in vitro* (LPS+INFg *vs* IL-4; GSE69607) or (**F**) *in vivo* (LPS+ *vs* LPS-; GSE38705) polarized macrophages (**G**). RT-qPCR analyses of *Arg1, Mrc1* and *Nos2* expression in non-treated, CTL-EVs or PTC-EVs treated BMDM (n=6). mRNA fold changes are normalized on the geometric mean of *Rpl27* and *Gapdh* expression. Data are expressed as mean ± SD; differences are not significant. (**H-J**) Concentration of TNFα, IL-6 and IL-10 released in BMDM medium after 24h of treatment with CTL-EVs and PTC-EVs compared to the basal production level in non-treated BMDM (dotted line).

Supplementary Table S1: Sequences of primers used in mRNAs RT-qPCR.

Gene	Forward primer	Reverse primer
Fosl1	CCGGCCAGGAGTCATACG	CTGCTAGCTTGTTCCGCTCG
Dusp5	CCAGTGTGGAAAGCCCGTT	CGCACTTGGATGCGTGGTA
Pax8	CGGCGATGCCTCACAACTCG	CCGGATGCTGCCAGTCTCGT
Nkx2.1	ATCTGAGCTGGGGTGCTGGG	GCCCTGTCTGTACGCTGCGA
Nis	AGCAGGCTTAGCTGTATCCC	AGCCCCGTAGTAGAGATAGGAG
Tg	TGGGACGTGAAAGGGGAATGGTGC	GTGAGCTTTTGGAATGGCAGGCGA
Tpo	TGCCAACAGAAGCATGGGCAAC	GCACAAAGTTCCCATTGTCCAC
Tshr	CTGCGGGGCAAAGAGTGTGC	AGGGGAGCTCTGTCAAGGCA
Rpl27	GCCCTGGTGGCTGGAATTGACC	AAACTTGACCTTGGCCTCCCGC
Gapdh	TGCACCACCAACTGCTTAGC	GGATGCAGGGATGATGTTCT

Supplementary Table S2: List of antibodies used.

**Antibodies used in western blot**					
**Antibody**	**Isotype**	**Clone**	**Supplier**	**Reference**	**Working dilution**
**Calnexin**	Rabbit	polyclonal	Enzo	ADI-SPA-860	1:10000
**PDI**	Rabbit	C81H6	Cell signaling technologies	3501	1:1000
**CD63**	Rat	NVG-2	Biolegend	143902	1:500
**CD81**	Rabbit	D502Q	Cell signaling technologies	10037	1:400
**CD9**	Rat	KMC8.8	eBiosciences	14-0091-82	1:200
**Alix**	Mouse	C-11	Santa Cruz	sc-271975	1:500
**Flotillin-1**	Mouse	18/Flotillin-1	BD transduction	610820	1:500
**Antibody used in immunohistofluorescence**					
**Antibody**	**Isotype**	**Clone**	**Supplier**	**Reference**	**Working dilution**

**CD206**	Rabbit	E6T5J	Cell signaling technologies	24595	1:500
**Fluorochrome-conjugated antibodies for FACS, flow cytometry and ExoView experiments**					
**Antibody**	**Fluorochrome**	**Clone**	**Supplier**	**Reference**	**Working concentration**
**CD3**	Alexa 700	17A2	biolegend	100216	10 µg/ml
**CD11b**	BV510/FITC	M1/70	biolegend	101263/101205	2 µg/ml
**CD31**	PE	MEC13.3	BD biosciences	553373	2 µg/ml
**CD45**	APC-Cy7	30-F11	BD biosciences	557659	1 µg/ml
**Ecad**	Alexa 647	DECMA-1	biolegend	147308	15 µg/ml

Supplementary Table S3: Sequences of primers and probes used in miRNAs RT-qPCR. Sequence of Universal reverse pimer is GGTGTCGTGGAGTCGGCAA

miRNA ID	Sequence	Loop-Reverse primer	Forward primer	Probe
mmu-miR-31-5p	AGGCAAGAUGCUGGCAUAGCUG	CTCAACTGGTGTCGTGGAGTCGGCAATTCAGTTGAGCAGCTATG	ACACTCCAGCTGGGAGGCAAGATGCTG	TCAGTTGAGCAGCTATG
mmu-miR-146b-5p	UGAGAACUGAAUUCCAUAGGCU	CTCAACTGGTGTCGTGGAGTCGGCAATTCAGTTGAGAGCCTATG	ACACTCCAGCTGGGTGAGAACTGAATTC	TCAGTTGAGAGCCTAT
mmu-miR-21a-5p	UAGCUUAUCAGACUGAUGUUGA	CTCAACTGGTGTCGTGGAGTCGGCAATTCAGTTGAGTCAACATC	ACACTCCAGCTGGGTAGCTTATCAGACT	TCAGTTGAGTCAACATC
mmu-miR-222-3p	AGCUACAUCUGGCUACUGGGU	CTCAACTGGTGTCGTGGAGTCGGCAATTCAGTTGAGACCCAGTA	ACACTCCAGCTGGGAGCTACATCTGGC	TCAGTTGAGACCCAGT
mmu-miR-221-3p	AGCUACAUUGUCUGCUGGGUUUC	CTCAACTGGTGTCGTGGAGTCGGCAATTCAGTTGAGGAAACCCA	ACACTCCAGCTGGGAGCTACATTGTCT	TCAGTTGAGGAAACCCA
mmu-miR-26a-5p	UUCAAGUAAUCCAGGAUAGGCU	CTCAACTGGTGTCGTGGAGTCGGCAATTCAGTTGAGAGCCTATC	ACACTCCAGCTGGGTTCAAGTAATCCA	TCAGTTGAGAGCCTATC
mmu-miR-204-5p	UUCCCUUUGUCAUCCUAUGCCU	CTCAACTGGTGTCGTGGAGTCGGCAATTCAGTTGAGAGGCATAG	ACACTCCAGCTGGGTTCCCTTTGTCAT	TCAGTTGAGAGGCATAG
mmu-miR-148a-5p	AAAGUUCUGAGACACUCCGACU	CTCAACTGGTGTCGTGGAGTCGGCAATTCAGTTGAGAGTCGGAG	ACACTCCAGCTGGGAAAGTTCTGAGAC	TCAGTTGAGAGTCGGAG
mmu-miR-148a-3p	UCAGUGCACUACAGAACUUUGU	CTCAACTGGTGTCGTGGAGTCGGCAATTCAGTTGAGACAAAGTT	ACACTCCAGCTGGGTCAGTGCACTACA	TCAGTTGAGACAAAGTT
mmu-miR-741-3p	UGAGAGAUGCCAUUCUAUGUAGA	CTCAACTGGTGTCGTGGAGTCGGCAATTCAGTTGAGTCTACATA	ACACTCCAGCTGGGTGAGAGATGCCAT	TCAGTTGAGTCTACATA
mmu-miR-30d-5p	UGUAAACAUCCCCGACUGGAAG	CTCAACTGGTGTCGTGGAGTCGGCAATTCAGTTGAGCTTCCAGT	ACACTCCAGCTGGGTGTAAACATCCCC	TCAGTTGAGCTTCCAGT
mmu-miR-99a-5p	AACCCGUAGAUCCGAUCUUGUG	CTCAACTGGTGTCGTGGAGTCGGCAATTCAGTTGAGCACAAGAT	ACACTCCAGCTGGGAACCCGTAGATCC	TCAGTTGAGCACAAGAT
mmu-miR-10b-5p	UACCCUGUAGAACCGAAUUUGUG	CTCAACTGGTGTCGTGGAGTCGGCAATTCAGTTGAGCACAAATT	ACACTCCAGCTGGGTACCCTGTAGAAC	TCAGTTGAGCACAAATTC
mmu-let-7f-5p	UGAGGUAGUAGAUUGUAUAGUU	CTCAACTGGTGTCGTGGAGTCGGCAATTCAGTTGAGAACTATAC	ACACTCCAGCTGGGTGAGGTAGTAGAT	TCAGTTGAGAACTATAC
mmu-miR-126a-3p	UCGUACCGUGAGUAAUAAUGCG	CTCAACTGGTGTCGTGGAGTCGGCAATTCAGTTGAGCGCATTAT	ACACTCCAGCTGGGTCGTACCGTGAGT	TCAGTTGAGCGCATTAT
mmu-let-7b-5p	UGAGGUAGUAGGUUGUGUGGUU	CTCAACTGGTGTCGTGGAGTCGGCAATTCAGTTGAGAACCACAC	ACACTCCAGCTGGGTGAGGTAGTAGGT	TCAGTTGAGAACCACAC
mmu-let-7c-5p	UGAGGUAGUAGGUUGUAUGGUU	CTCAACTGGTGTCGTGGAGTCGGCAATTCAGTTGAGAACCATAC	ACACTCCAGCTGGGTGAGGTAGTAGGT	TCAGTTGAGAACCATAC

Supplementary Table S4: List of the most frequently sequenced miRNAs in EVs isolated from control thyroids, with the associated count per million (CPM).

Name	Mean CPM
**miR-125b-5p**	213400
**let-7c-5p**	100233
**let-7b-5p**	83644
**let-7f-5p**	76620
**miR-125a-5p**	66392
**let-7a-5p**	58649
**miR-126a-3p**	40698
**miR-30d-5p**	37117
**miR-29a-3p**	35818
**miR-16-5p**	34094

**miR-148a-3p**	31128
**let-7i-5p**	29743
**miR-26a-5p**	23394
**miR-199a-3p**	19919
**miR-30a-5p**	16369
**miR-143-3p**	16316
**let-7e-5p**	13416
**miR-200a-3p**	11859
**miR-191-5p**	11082
**miR-26b-5p**	10077

Supplementary Table S5: List of the most frequently sequenced miRNAs in EVs isolated from doxycycline-treated thyroids after 2 days of injections, with the associated count per million (CPM).

Name	Mean CPM
**miR-125b-5p**	147446
**let-7f-5p**	75838
**let-7b-5p**	73932
**let-7c-5p**	72227
**let-7a-5p**	56754
**miR-126a-3p**	43233
**miR-125a-5p**	42283
**let-7i-5p**	39875
**miR-16-5p**	39776
**miR-29a-3p**	37769
**miR-148a-3p**	30527
**miR-30d-5p**	25504
**miR-21a-5p**	21269
**miR-199a-3p**	20696
**miR-26a-5p**	19705
**miR-143-3p**	19673
**miR-30a-5p**	15666
**let-7e-5p**	14275
**miR-221-3p**	10761
**miR-200a-3p**	10619

Supplementary Table S6: List of the most frequently sequenced miRNAs in EVs isolated from doxycycline-treated thyroids after 4 days of injections, with the associated count per million (CPM).

Name	Mean CPM
**miR-125b-5p**	113447
**miR-126a-3p**	52815

**let-7b-5p**	52375
**let-7c-5p**	48836
**miR-16-5p**	47741
**let-7f-5p**	47140
**miR-21a-5p**	41538
**let-7a-5p**	38777
**let-7i-5p**	38526
**miR-125a-5p**	26307
**miR-29a-3p**	24603
**miR-199a-3p**	15656
**miR-143-3p**	13213
**miR-221-3p**	13163
**miR-30d-5p**	12642
**let-7e-5p**	11214
**miR-26a-5p**	11184
**miR-191-5p**	9513
**miR-146b-5p**	8998
**miR-23a-3p**	8789

Supplementary Table S7: List of miRNAs with a differential abundance, expressed in logFC, in EVs isolated at day 2 and day 4, as compared to control thyroids. MiRNAs marked with a "*" also present a differential abundance in the day 4 and day 2 samples.

Name	2*24h vs CTL	4*24h vs CTL
**miR-31-5p ***	4.127	5.472
**miR-135b-5p ***	2.830	3.913
**miR-21a-3p**	3.110	3.643
**miR-21a-5p ***	2.465	3.466
**miR-146b-5p**	3.102	3.360
**miR-155-5p ***	1.584	3.081
**miR-223-5p**	2.426	3.066
**miR-5099**	2.046	2.970
**miR-223-3p ***	1.783	2.924
**miR-142a-5p ***	1.354	2.669
**miR-20a-5p**	1.914	2.582
**miR-142a-3p ***	1.574	2.533
**miR-184-3p**	1.877	2.263
**miR-221-3p**	1.874	2.207
**miR-218-5p**	1.655	1.917
**miR-484**	1.477	1.843

**miR-674-5p**	1.270	1.647
**miR-125b-1-3p ***	0.825	1.646
**miR-222-3p**	1.220	1.511
**miR-125a-5p**	-0.665	-1.303
**miR-206-3p**	-1.365	-1.490
**miR-200c-3p ***	-0.639	-1.786
**miR-204-5p ***	-1.040	-2.773
